# Morphological change of CD4^+^ T cell during contact with DC modulates T-cell activation by accumulation of F-actin in the immunology synapse

**DOI:** 10.1186/s12865-015-0108-x

**Published:** 2015-08-26

**Authors:** Wei Lin, Yuanzhen Suo, Yuting Deng, Zhichao Fan, Yijie Zheng, Xunbin Wei, Yiwei Chu

**Affiliations:** Department of Immunology and Key Laboratory of Medical Molecular Virology of MOE/MOH, School of Basic Medical Sciences, Fudan University, Shanghai, 200032 China; Med-X Research Institute and School of Biomedical Engineering, Shanghai Jiao Tong University, 1954 Huashan Road, Shanghai, 200030 China; Biotherapy Research Centre, Fudan University, 138 Yixueyuan Road, Shanghai, 200032 China

**Keywords:** T-cell activation, F-actin, Immunological synapse, Calcium signal

## Abstract

**Background:**

The changes in T-cell morphology during immunological synapse (IS) formation are essential for T-cell activation. Previous researches have shown that T cell changed from spherical to elongated and/or flattened during in contact with B cell. As most powerful antigen presenting cell, dendritic cell (DC) has a strong ability to activate T cells. However, the morphological change of T cell which contacts DC and the relationship between morphological change and T-cell activation are not very clear. Thus, we studied the morphological change of CD4^+^ T cell during contact with DC.

**Results:**

Using live-cell imaging, we discovered diversity in the T-cell morphological changes during contact with DCs. The elongation-flattening of CD4^+^ T cells correlated with a low-level Ca^2+^ response and a loss of T-cell receptor (TCR) signalling molecules in the IS, including zeta-chain associated protein kinase 70 (ZAP-70), phospholipase C-γ (PLC-γ) and protein kinase C-θ (PKC-θ), whereas rounding-flattening correlated with sufficient CD4^+^ T-cell activation. Different morphological changes were correlated with the different amount of accumulated filamentous actin (F-actin) in the IS. Disruption of F-actin by cytochalasin D impaired the morphological change and the localisation of calcium microdomains in the IS and decreased the calcium response in CD4^+^ T cells.

**Conclusion:**

Our study discovered the diversity in morphological change of T cells during contacted with DCs. During this process, the different morphological changes of T cells modulate T-cell activation by the different amount of F-actin accumulation in the IS, which controls the distribution of calcium microdomains to affect T-cell activation.

**Electronic supplementary material:**

The online version of this article (doi:10.1186/s12865-015-0108-x) contains supplementary material, which is available to authorized users.

## Background

T-cell activation is central to the adaptive immune response. The first step in T-cell activation is immunological synapse (IS) formation at the interface of a T cell and an antigen-presenting cell (APC) during antigen recognition. The IS is a multi-molecular assembled of receptors and adhesion molecules, consisting of a central cluster of T-cell receptors (TCRs) that form the central supramolecular activation cluster (c-SMAC) and a surrounding ring of adhesion molecules (the peripheral SMAC, or p-SMAC) [1, 2]. During synapse formation, molecular re-organisation of plasma membrane proteins occurs in concert with a complex and dramatic morphological change in the cell, which is primarily driven by active cytoskeletal processes, such as actin cytoskeleton rearrangements and microtubule-organising centre (MTOC) reorientation [3–6]. The cytoskeleton rearrangements result in an altered morphology, including cell polarisation, which is essential for T-cell activation. However, not all antigen-specific T cells are sufficiently activated by contact with an APC [7, 8], so the factors affecting the morphological changes in and activation of T cells have attracted much attention.

The morphology of T cell has been reported to change from spherical to elongated and/or flattened in a dynamic manner during IS formation [8–10]. The elongated morphology is believed to be a characteristic of a mobile T cell which is scanning or spreading toward APCs, whereas the flattened T cell is immobile and forms stable contact with the APC [8, 10]. The process of a T cell encountering an antigen-presenting B cell lasts >20 min and consists of three distinct stages: contact, recognition, and stabilisation [11]. The contact phase lasts from 20 s to several minutes and consists of T-cell probing of and contact with the B cell prior to any change in [Ca^2+^]_i_. The recognition phase lasts 1–3 min and is the most dynamic stage of the interaction, coupled with a [Ca^2+^]_i_ increase. The third phase (stabilisation) occurs after [Ca^2+^]_i_ relaxes from the peak level and is morphologically characterised by partial retraction and subsequent rounding of the T cell [11]. The dendritic cell (DC) is the most powerful APC and has a strong ability to activate T cells. The type of synapse formed between a T cell and a DC is different from that between a T cell and a B cell [12, 13], which may reflect different cytoskeleton rearrangements and morphological changes in a T cell in contact with a DC. However, the morphological change of T cell during contact with DC is not very clear.

Filamentous actin (F-actin) network plays a central role in IS formation, morphological change and TCR signalling [14]. Treatment of T cells with actin depolymerising agents leads to loss of Ca^2+^ mobilization and downstream transcriptional activation [15]. A necessary step for the activation of T cells following TCR engagement is the stimulation of Ca^2+^ entry across the plasma membrane [16, 17]. TCR activation causes a series of signalling molecules to accumulate in the IS, such as zeta-chain-associated protein kinase 70 (ZAP-70), linker of activated T cells (LAT) and phospholipase C-γ (PLC-γ). The activity of PLC-γ generates inositol 1,4,5-trisphosphate, which releases Ca^2+^ from the endoplasmic reticulum (ER) and promotes activation of the Ca^2+^ release-activated Ca^2+^ (CRAC) channels encoded by the ORAI proteins in the plasma membrane [6, 18]. The Ca^2+^ influx through the CRAC/ORAI channels triggers the translocation of mitochondria towards the plasma membrane or IS, and these mitochondria in turn maintain calcium channel activity and thereby allow more Ca^2+^ influx to induce global cytosolic Ca^2+^ signalling [17, 19, 20]. When mitochondrial accumulation at the IS is prevented, Ca^2+^ accumulates in a microdomain close to the CRAC/ORAI channels, leading to inactivation of the channels and efficient Ca^2+^ clearance by plasma membrane Ca^2+^ ATPases (PMCAs). Therefore, the global cytosolic Ca^2+^ concentration becomes low [20]. Cytoskeletal networks have been reported to affect intracellular Ca^2+^ signalling by controlling the spatial and temporal distribution of Ca^2+^ sources and sinks and by modulating TCR-dependent Ca^2+^ signals, which are required for an appropriate T-cell response [6, 21].

In our study, we imaged the diversity of morphological changes in antigen-specific T cells during contact with DCs. We found that a relatively round and flattened T-cell shape was correlated with a strong and sustained Ca^2+^ response in the T cell, whereas an mobility and elongated-flattened T-cell shape was correlated with a limited Ca^2+^ response. Furthermore, morphological changes modulated T-cell activation by the accumulation of F-actin which controls the distribution of calcium microdomains. The accumulation of F-actin in the IS plays an important role in T-cell activation.

## Methods

### Animals and cell culture

OT-II transgenic mice (C57BL/6 background) were gifts from Professor Yanmei Han (The Second Military Medical University, Shanghai, China) and were purchased from the Jackson Laboratory (Bar Harbor, Maine, ME, USA). All animals were maintained in a specific pathogen-free facility. All animal experiments were approved by the Ethics Committee of Fudan University (20120302–023) and were undertaken in accordance with the Guidelines for the Care and Use of Laboratory Animals (No. 55 issued by Ministry of Health, Peoples Republic of China on January 25th, 1998). Primary CD4^+^ T cells were obtained from the spleen of 6–8 week OT-II × Rag−/− mouse. OT-II CD4^+^ T cells were selected using a CD4 negative-selection kit (Miltenyi Biotech, Auburn, CA, USA). Cells were cultured in RPMI 1640 (Sigma, St. Louis, MO, USA) containing 10 % FBS (Atlanta Biologicals, Atlanta, GA, USA) at 37 °C in 5 % CO_2_. For primary T cell culture, cells were maintained in RPMI 1640 containing 10 % FBS and supplemented with 1 mM L-glutamine, 50 μM 2-mercaptoethanol (2-ME), and 1 mM non-essential amino acids. The DC2.4 cell line was a kind gift from Professor Kenneth L. Rock (University of Massachusetts Medical School) and cultured in RPMI 1640 containing 10 % FBS at 37 °C in 5 % CO_2._ ICAM-1-EGFP/DC2.4 cell line was constructed and cultured as previous report [13].

### Reagents and antibody

The reagents used in our experiments included: OVA_(323–339)_ (Sigma-Aldrich, St Louis, MO, USA), thapsigargin (TG, stock 1 mM in DMSO, Molecular Probes, Invitrogen, USA), cytochalasin D and nocodazole (Calbiochem, Merck KGaA, Germany). Recombinant murine interferon gamma (IFN-γ) was purchased from Peprotech (Rocky Hill, NJ, USA). For live cell imaging, H57-597-Fab-TCRαβ-Alexa Fluor 647 was purchased from Invitrogen to label TCR as a non-blocking antibody [22]. For calcium imaging, Calcium Crimson™ was purchased from Invitrogen. The following antibodies (Abs) were used for immunofluorescence: Texas Red-X phalloidin for anti-F-actin, MitoTracker® Green FM (Molecular Probes, Invitrogen, USA), anti-PLCγ1 (sc81, Santa Cruz Biotechnology, Santa Cruz, CA, USA), polyclonal rabbit anti-PKC-θ (sc212, Santa Cruz Biotechnology), anti-ZAP-70 (Y319, Abcam, Cambridge, MA, USA), anti-ORAI1 (ab59330, Abcam, Cambridge, MA, USA) and anti-PMCA (5 F10, Abcam, Cambridge, MA, USA), Dylight 405-conjugated goat anti-rabbit IgG (Jackson ImmunoResearch Laboratories), Alexa Fluor 555-conjugated goat anti-rabbit IgG (Invitrogen). The following Abs were used for flow cytometry: anti-MHC-II (I-Ab)-FITC, antiCD80-PE, anti-CD86-APC and anti-CCR7-APC (all from eBioscience).

### DC maturation and CD4^+^ T cell-DC contact

For ICAM-1-EGFP/DC2.4 cell maturation, IFN-γ (20 ng/ml) was added into the medium for overnight incubation. Then LPS (lipopolysaccharide, 100 ng/ml) was added for one hour incubation [23, 24]. OVA_(323–339)_ (5 μM) was used to pulse the DC cell line for 2-h. OT-II CD4^+^ T cells labelled with Alexa Fluor 647-conjugated H57 antibody were added to matured DC (T: DC = 10:1) for live cell imaging. For some experiments, T cells were pre-incubated at 37 °C with 1 μM, 2.5 μM, 5 μM or 10 μM cytochalasin D for 30 min, or 2 μM nocodazole for 16 h. Those T cells were washed by PBS buffer, and then added to OVA_(323–339)_-pulsed DCs. For TG stimulation, TG (1 μM) was used to stimulate T cells for 1 min to image or add to OVA_(323–339)-_pulsed DCs.

### Confocal microscopy

Images were taken with a confocal microscope (Leica TCS SP5, Leica Microsystems GmbH, Wetzlar, Germany) equipped with an APO oil immersion objective lens (63×, NA = 1.40). To quantify redistribution of molecules at the contact site, T-DC doublets were chosen from bright-field images and evaluated by the fluorescence image stacks. Image stacks consisted of 6–15 sections with 1 μm per step at *z* axis. Time-lapse scanning was used for live cell imaging for 30–60 min with 512 × 512 pixels per frame and 40 or 10 s as the interval.

### Ca^2+^ imaging

For Ca^2+^ imaging, OT-II CD4^+^ T cells were incubated with H57-Fab-TCRαβ-Alexa Fluor 647 for 30 min at 4 °C, washed twice, then labelled with 10 μM Calcium Crimson™ in 1 mL calcium free PBS for 60 min at 25 °C. Then the cells were washed two times, and were added to OVA_(323–339)_-pused ICAM-1-EGFP/DC2.4. Afterwards, the cells were maintained throughout the experiment in mammalian Ringer solution containing (in mM): 160 NaCl, 4.5 KCl, 2 CaCl_2_, 1 MgCl_2_, 10 Hepes (pH = 7.4; osmolality 290–310 milliosmoles/kg), supplemented with 11 mM glucose. Calibration was performed by measuring fluorescence intensities in the absence of calcium (*F*_min_) or the calcium-saturated solution (*F*_max_), and applying the equation of *Takahashi et al.* [25] *F*_min_ was established at 1 mM EGTA with calcium-free solution. *F*_max_ was established at 1 mM ionomycin with 10 mM Ca^2+^. Ca^2+^ images were taken every 5–20 min after adding T cells to the DCs. The scans were made at 10-s or 40-s intervals to determine whether calcium concentration rose in the T cells after contacting DCs. Then *z*-planes scanning were used to determine the synapse structure.

### Immunofluorescence staining

For ZAP-70 and other intracellular molecule staining, after the T cells had contacted DCs for 30 mins, all the cells were fixed in PBS/4 % paraformaldehyde for 10 min, followed by PBS/0.1 M glycine incubation for 3 min, as reported previously [26]. Cells were permeabilized with PBS/0.1 % Triton X-100 for 20 min, and then blocked with PBS/2 % BSA buffer for 20 min. Afterwards, the cells were stained with a 1:10 dilution of anti-ZAP-70 antibody for 60 min. After being washed three times, the secondary antibody was diluted 1:100 to 1:400 and incubated with cells for 30 min. The images of cells were observed with a confocal microscope. For F-actin staining or mitochondrial labeling, the antibody of F-actin or MitoTracker was used as previously reported [27].

### Imaging analysis

The images were analysed with the Leica Application Suite Advanced Fluorescence software (Leica TCS SP5, Leica Microsystems GmbH, Wetzlar, Germany or the Imaris software (Bitplane AG, Zurich, Switzerland). Segmentation, rendering, area, spot tracking and maximum intensity projection were used for data visualization analysis. Utilizing these tools, 3D intercellular contacts could be visualized from multiple angles. Only T-DC pairs whose contact orientation was proper for *x-y* plane projection were taken into consideration for further analysis.

A quantitative estimation of morphological change was obtained by calculating the shape index: shape index = P^2^/4πS [10]. The P and S are the perimeter and the area of the cross section of a cell (may be a regular circle or an irregular circle) respectively. These values were calculated from a semiautomatic definition of the outline of the cell, obtained with Imaris software. When the planar projection of a cell (like a disk or a sphere) is a circle, the shape index is approximately 1. Any departure from a circle gives a shape index > 1, reflecting the cell was elongated [8, 10]. We defined a cell as a round cell if the shape index was within 0.8-1.3, and defined a cell as an elongated cell if the shape index was above 1.3.

**Fig. 1:**
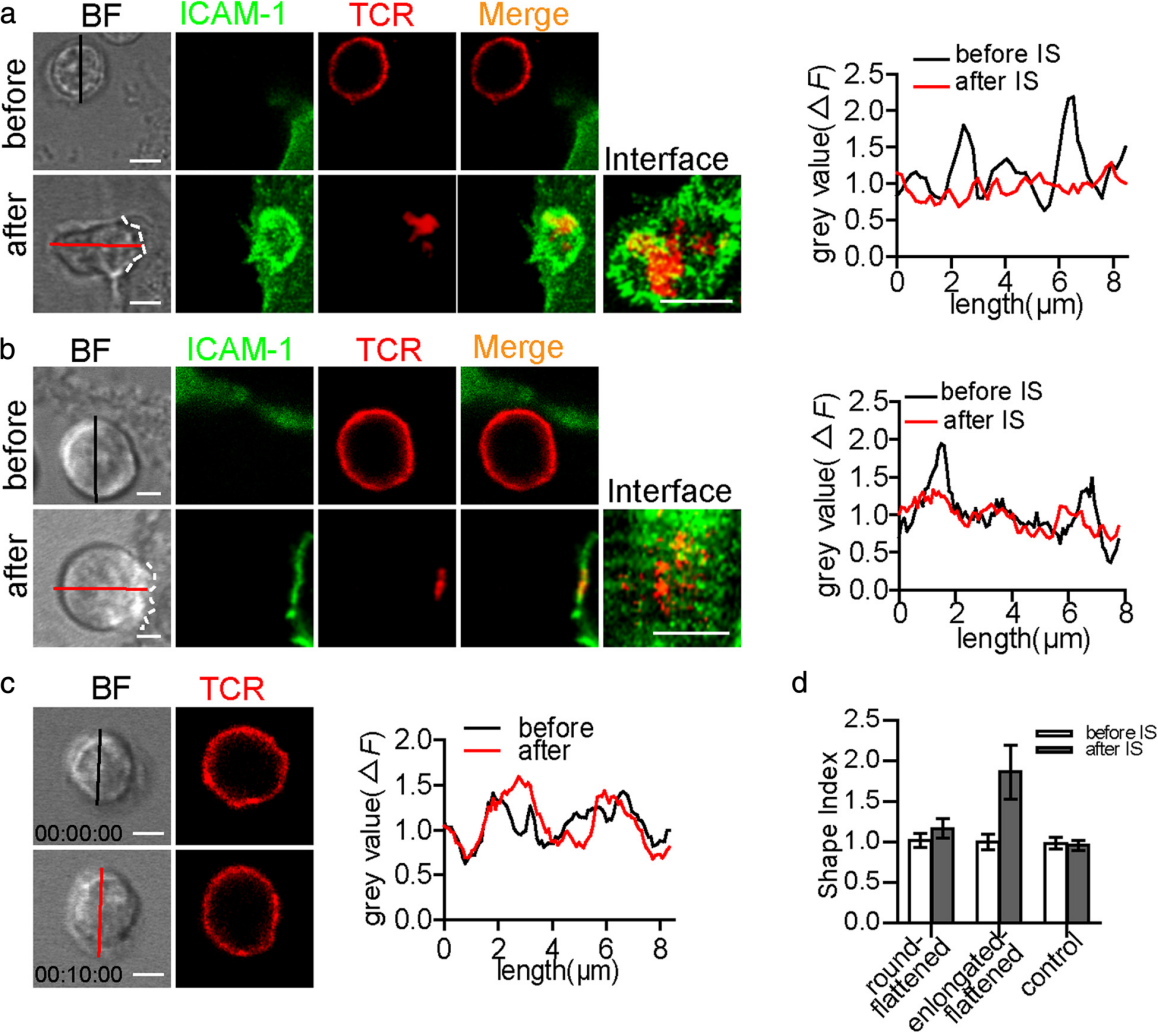
Morphological changes in T cells following IS formation. **a** *Left panel*: An elongated-flattened CD4^+^ T cell before and after T cell-DC contact. TCRs were labelled with H57-597-Fab-Alexa 647 (red), and ICAM-1 was fused with EGFP (green). *Right panel*: Grey value profiles along the red and black lines in the BF image. The black line is before contact, and the red line is after contact. **b** A round-flattened CD4^+^ T cell before and after T cell-DC contact (left panel) and the grey value analysis (right panel). In (**a**) and (**b**), the last column in the left panel is 3D images of the contact surface which consisted of ICAM-1 (green) surrounding TCRs (red). The dotted white line depicts the contact boundary between the CD4^+^ T cells and the DCs. **c** A resting CD4^+^ T cell (left panel) at 0 min (top line) and 10 min (bottom line). The right panel depicts the grey value profiles along the red and black lines. Scale bar = 2 μm. **d** Comparison of the shape index of elongated and/or flattened CD4^+^ T cells and control CD4^+^ T cells before and after T cell-DC contact (round-flattened: 1.01 ± 0.10 vs 1.16 ± 0.18, elongated-flattened: 1.00 ± 0.10 vs 1.86 ± 0.53, control: 1.00 ± 0.10 vs 0.98 ± 0.12). In total, 25 cells were analysed in each group (the data are shown as the mean ± s.e.m.)

The flattened morphology change was measured by the contrast change between the edges and the middle part along a line (Fig. 1) according to a previous report [27]. Briefly, the flattening of a cell correlated with a reduction of the contrast between the edge (mostly plasma membrane) and the middle part (mostly intracellular) of the cell when analysed by the gray value of the bright field (BF) image. Then we defined a cell which became elongated and flattened as an elongated-flattened cell and define a cell which only became flattened as a round-flattened cell.

The contact area was assumed to form a spherical cap with a solid angle of 2α on the T cell. It is estimated by the equation *C* = 2π*R*^2^ (1 - cosα) [7], where C is the contact area while α is half of the solid angle for the contact cap.

### Data analysis

Data analysis was performed with GraphPad Prism 5 (GraphPad Software, San Diego, CA). For all statistics, two-tailed Student’s *t*-test and one-way ANOVA were applied to compare two normal distribution datasets. Mann–Whitney *U* test was used to compare two nonparametric datasets. Significance levels and symbols employed were *p* < 0.05 (*), *p* < 0.01 (**) and *p* < 0.001(***).

## Results

### Two different types of morphological changes in CD4^+^ T cells were analysed during IS formation

To investigate the morphological changes in CD4^+^ T cells during IS formation, we sorted splenic CD4^+^ T cells from OT-II transgenic mice and labelled the TCR clusters. Additionally, ICAM-1-EGFP was transfected into the DC2.4 cell line to show the IS structure. After the CD4^+^ T cells were placed in contact with OVA_(323–339)_-pulsed DCs, the synapse structure was measured using confocal microscopy. We found that only those CD4^+^ T cells forming a stable synapse became flattened (Fig. 1a-b, right panel). Using a protocol from a previous report [27], the flattening was measured based on the contrast change between the edges and the middle part along a line. A naive T cell is spherical in shape before it makes contact with an APC. After the T cell made contact with a DC, the T cell changed to a flattened morphology, with the contrast between the edges and the middle part reduced to approximately 1 (the grey value was measured using bright-field (BF) microscopy, Fig. 1a-b right panel). However, those CD4^+^ T cells that interacted with DCs transiently or that scanned DCs without forming a synapse did not become flattened (Additional file 1: Figure S1A and C). These findings indicated that the flattened shape was correlated with IS formation.

For the CD4^+^ T cells that interacted with DCs, 15 % of T cells changed from spherical to elongated and flattened in shape (Fig. 1a, Additional file 2: Figure S2A and Additional file 3: Video 1; the shape index changed from 1 to 2.5). Neither immobility nor round shape was observed among these T cells during IS formation (Additional file 3: Video 1). In contrast, 52 % of the CD4^+^ T cells in contact with DCs became flattened and remained round during IS formation (Fig. 1b, Additional file 2: Figure S2B and Additional file 4: Video 2). The shape index of the round-flattened T cells slightly fluctuated around 1 during the process of T cell-DC contact. During this process, certain T cells extended their pseudopodia to make contact with DCs in several frames (Additional file 4: Video 2). Other T cells were scanning T cells and did not become flattened during contact with DCs (Additional file 1: Figure S1A and C). The duration of the elongated-flattened and round-flattened CD4^+^ T cells’ contact with DCs was more than 1 h, indicating that both morphological changes in the CD4^+^ T cells allowed the formation of a stable synapse with DCs.

In the absence of antigen, the percentage of T cell-DC contact decreased significantly. Nearly no stable T cell-DC contact was found. Neither the round-flattened nor the elongated-flattened change was observed among the CD4^+^ T cells. As the antigen concentration increased, the number of elongated-flattened and round-flattened T cells increased, whereas the ratio of round-flattened T cells to elongated-flattened T cells among all T cell-DC conjugates was not changed (Additional file 5: Figure S3).

### The elongated-flattened morphological change correlated with ineffective T-cell activation, whereas the round-flattened morphological change correlated with effective T-cell activation

**Fig. 2:**
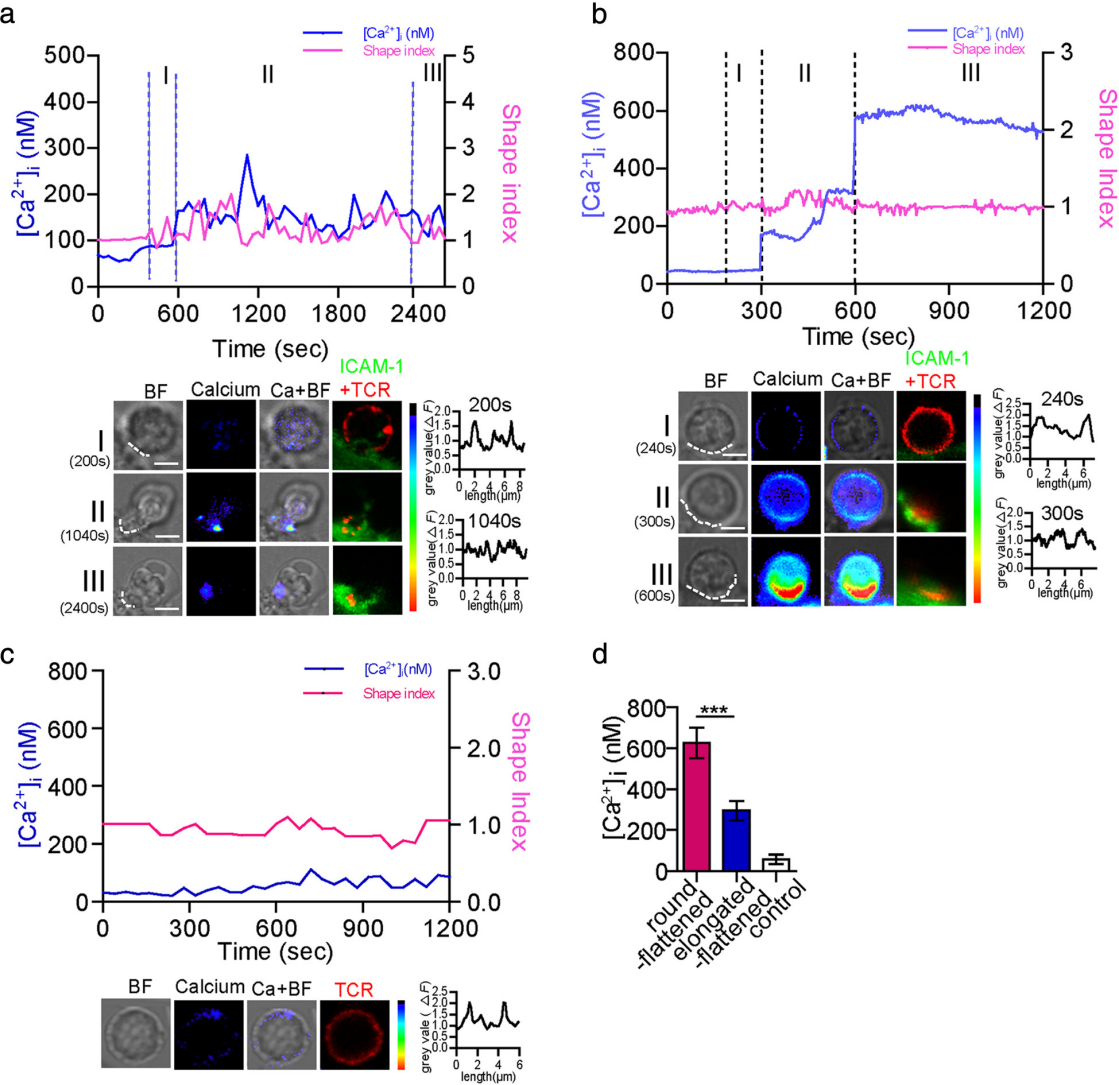
Morphological changes correlated with sustained Ca^2+^ signals in CD4^+^ T cells. **a** Shape index changes and Ca^2+^ signals in a CD4^+^ T cell whose morphology changed to elongated-flattened (top panel). Phases I, II, and III refer to contact, recognition, and interaction, respectively. Images of the morphology and Ca^2+^ signals of the elongated-flattened T cell in the three phases are shown in the left panel at the bottom. The contrast between the edges and the middle of the cell changed at 200 s and 1040 s (right column of the left-hand bottom panel). **b** Shape index changes and Ca^2+^ signals in a CD4^+^ T cell whose morphology changed to flattened (top panel) during the three phases. Images of the morphology and Ca^2+^ signals of the round-flattened T cell in the three phases are shown in the left panel at the bottom. The contrast between the edges and the middle part of the cell changed at 240 s and 300 s (right column of the left-hand bottom panel). The dotted white line depicts the contact boundary between the OT-II CD4^+^ T cells and the DCs. **c** Shape index changes and Ca^2+^ signals in a single resting CD4^+^ T cell. The contrast between the edges and the middle of the cell changed at 600 s (right column of the left-hand bottom panel). (A-C) Ca^2+^ signalling was observed every 10 s or 40 s. The calcium signal intensity was pseudo-colored with hues ranging from blue (low) to red (high). ICAM-1 was labelled to be green, TCR was labelled be red. Before IS formation, TCR was uniformed on the surface of T cell. After IS formation, TCR and ICAM-1 were accumulated into the IS of DC-T. **d** Average Ca^2+^ responses in CD4^+^ T cells whose morphology changed to round-flattened (625±74 nM, *n*=50) or elongated-flattened (294±46 nM, *n*=50). The average Ca^2+^ response in the resting CD4^+^ T cells was 57±23 nM (n=50; the data are shown as the mean ± s.e.m., two-tailed Student’s t-test, ***P<0.001). Scale bar = 2 μm

Previous reports showed that only 13 % and 60 % of antigen-specific T cells could be activated by B cells and DCs, respectively [8]. To investigate whether the distinct morphologies might induce different T-cell activation states, we dynamically measured the morphological changes and calcium signals after an OT-II CD4^+^ T cell came into contact with an OVA_(323–339)_-pulsed DC. We found that the trigger of Ca^2+^ signal was correlated with the change to the flattened shape of T cells. The elongated-flattened morphology of the CD4^+^ T cells correlated with low-level Ca^2+^ signals. A Ca^2+^ signal was released in an elongated-flattened CD4^+^ T cell during contact with a DC (Fig. 2a and Additional file 6: Figure S4A). Before the OT-II CD4^+^ T cell made contact with the DC, its shape index slightly fluctuated around 1. The T cell then made contact with the DC, and its shape index changed, resulting in dramatic elongation during the contact phase (phase I, from 320 s to 600 s). TCRs began to move into the centre of the cell-cell contact (Fig. 2a). In contrast with T cell recognition of a B cell [11], this T cell needed a long time to recognise the DC (Fig. 2a, phase II, from 600 s to 2400 s). The T cell became flattened at 1040 s, after which the Ca^2+^ signal was triggered. The peak of the Ca^2+^ signal was 300 nM. In this phase, TCRs and ICAM-1 accumulated to form the synapse (Fig. 2a). After the recognition phase, the Ca^2+^ response decreased (Fig. 2a, phase III).

In contrast to the elongated-flattened morphological change, the round-flattened change in the CD4^+^ T cells correlated with a high level of Ca^2+^ signalling. A Ca^2+^ signal was released in a round-flattened CD4^+^ T cell during contact with a DC (the peak of the Ca^2+^ signal was 650 nM; Fig. 2b and Additional file 6: Figure S4B). The T cell made contact with the DC at 200 s and had no obvious morphological change. In the recognition phase (Fig. 2b, phase II, from 300 s to 600 s), the shape index of the round-flattened T cell fluctuated in the range of 0.8-1.3. The T cell became flattened at 300 s, and a Ca^2+^ signal was released at that moment (Fig. 2b and Additional file 6: Figure S4B). The Ca^2+^ signal was triggered at 600 s and sustained for at least 10 min. After the recognition phase, the shape index was approximately 1, and the Ca^2+^ response was sustained at a high level. The recognition phase of round-flattened T cell-DC contact was shorter than that of an elongated-flattened T cell. The mean peak of Ca^2+^ responses in the elongated-flattened CD4^+^ T cells were significantly lower than that in the round-flattened CD4^+^ T cells (294 ± 46 nM vs 625 ± 74 nM; Fig. 2d). The Ca^2+^ signals were not induced in resting or scanning CD4^+^ T cells (Fig. 2c and Additional file 1: Figure S1B and D). These results indicated that different morphological changes correlated with different T-cell activation states.

**Fig. 3:**
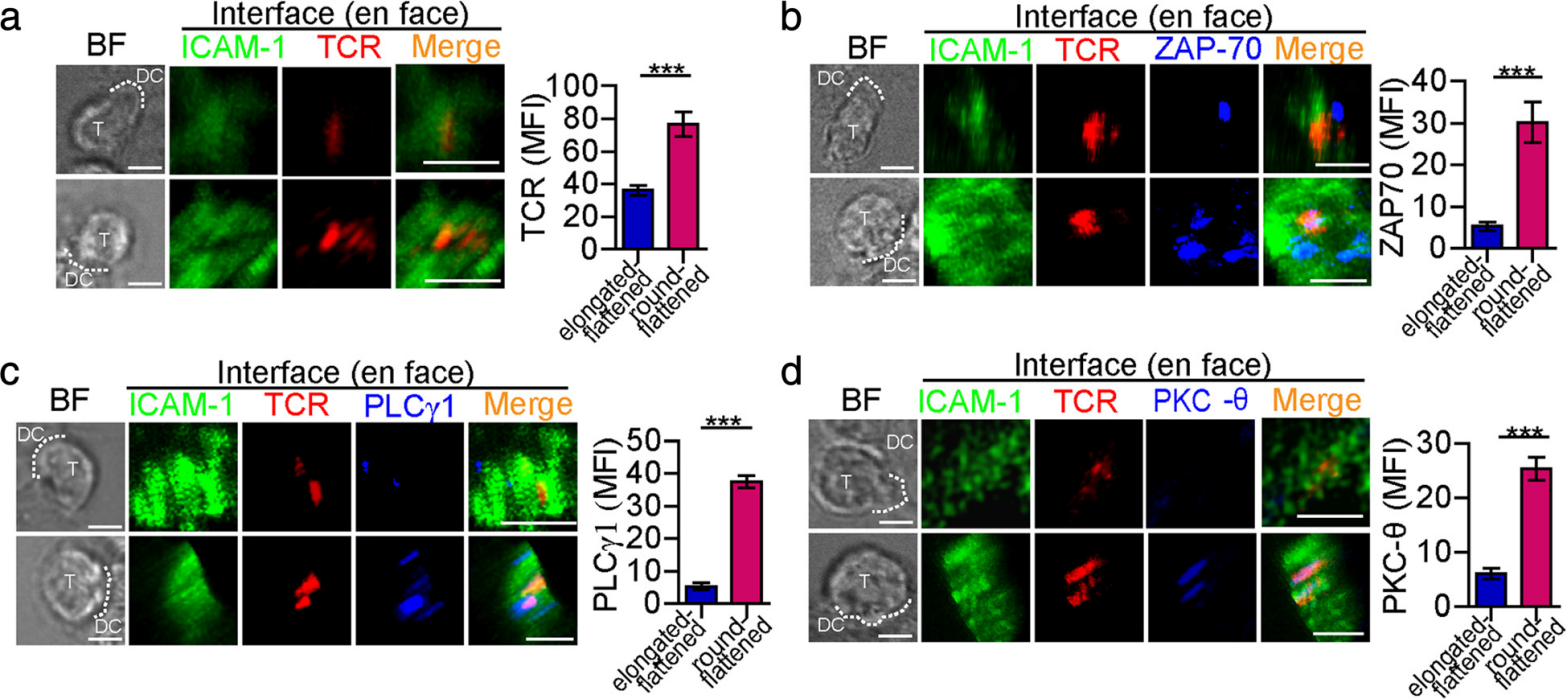
Morphological changes correlated with the localisation of TCR signalling molecules in the IS. **a**-**d** *Left pane*l: The localisations of TCR, ZAP-70, PLC-γ1, and PKC-θ (all in blue) were measured in a T cell becoming elongated-flattened (upper row) or round-flattened (bottom row) when IS formation. TCR (red) and ICAM-1 (green) were labelled to show the IS formation. The dotted white line depicts the contact boundary between the OT-II CD4^+^ T cells and the DCs. Scale bar = 2 μm. *Right panel*: A quantitative analysis was used to compare the MFIs of the T-cell signalling molecules in the IS of the round-flattened T cells and the elongated-flattened T cells (mean ± s.e.m., n=25, three independent experiments), ***P<0.001 (two-tailed Student’s t-test). Scale bar = 2 μm

To further investigate the correlation between the morphological changes and T-cell activation, TCR signalling pathway molecules were detected. Although TCRs accumulated in the IS in both types of morphologically changed CD4^+^ T cells, the mean fluorescence intensity (MFI) of the TCRs in the IS of the elongated-flattened CD4^+^ T cells was lower than that in the IS of the round-flattened CD4^+^ T cells (Fig. 3a). Furthermore, ZAP-70, a parameter that reflects the TCR’s downstream signal, was measured in the IS of elongated and/or flattened CD4^+^ T cells. ZAP-70 had a limited or no tendency to accumulate in the IS of elongated-flattened CD4^+^ T cells and did not co-localise with the TCR cluster. However, ZAP-70 was obviously recruited into and accumulated in the IS of round-flattened CD4^+^ T cells and co-localised with TCRs (Fig. 3b). The MFI of ZAP-70 in the IS of the elongated-flattened CD4^+^ T cells was lower than that in the IS of the round-flattened CD4^+^ T cells (Fig. 3b). PLC-γ1 and PKC-θ, the downstream signalling molecules of ZAP-70, did not co-localise with the TCR cluster in the IS of the elongated-flattened CD4^+^ T cells (Fig. 3c-d). In contrast, both of these molecules were present in the IS and co-localised with TCRs in the round-flattened CD4^+^ T cells. In the elongated-flattened T cells, ZAP-70 did not co-localise with TCRs, leading to impairment of ZAP-70 activation, which blocked downstream signalling.

Taken together, elongated-flattened CD4^+^ T cells may lose the ability to be activated due to a defect in TCR signalling molecule accumulation and may be induced to produce a low-level Ca^2+^ response, whereas round-flattened CD4^+^ T cells can be activated.

### The amount of F-actin accumulating in IS differed in different morphological change of CD4^+^ T cell

**Fig. 4:**
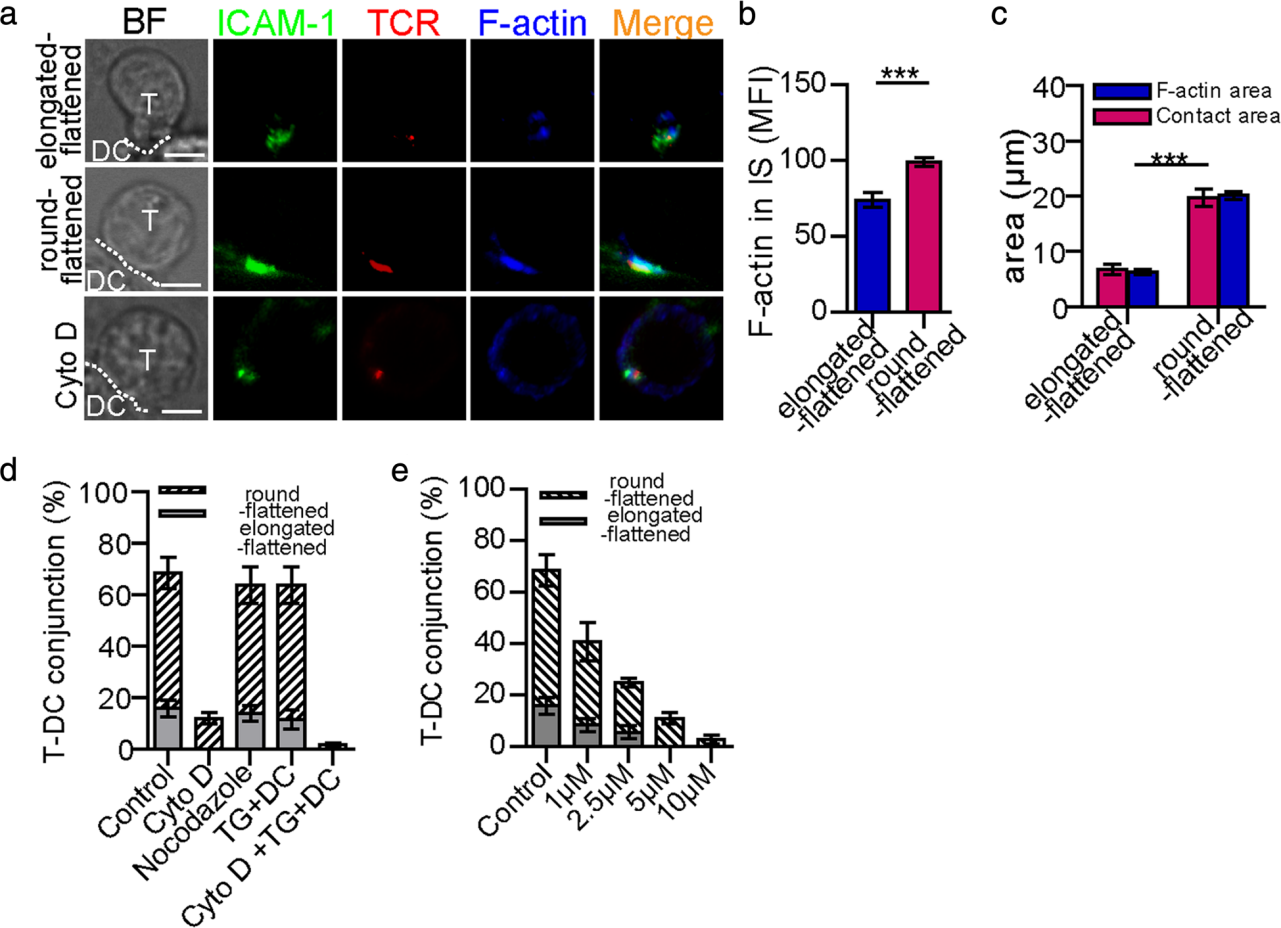
The morphological changes in CD4^+^ T cells were dependent on the F-actin cytoskeleton. **a** The accumulation of F-actin (blue) in an elongated-flattened CD4^+^ T cell (top line) or a round-flattened CD4^+^ T cell (middle line) after IS formation. The distribution of F-actin in a CD4^+^ T cell after it made contact with an OVA_323-339_-pulsed DC in the presence of cytochalasin D is also depicted. TCR (red) and ICAM-1 (green) were labelled to show the IS formation. The dotted white line depicts the contact boundary between the CD4^+^ T cells and the DCs. Scale bar = 2 μm. **b** The MFIs of F-actin in the IS of the round-flattened T cells and the elongated-flattened T cells (mean ± s.e.m., *n*=25, three independent experiments), ***P<0.001 (two-tailed Student’s t-test). **c** Comparison of the contact area of T cell-DC contact based on the area of accumulation of F-actin in the IS (mean ± s.e.m., *n*=25, three independent experiments), ***P<0.001 (two-tailed Student’s t-test). **d** The percentage of elongated and/or flattened CD4^+^ T cells was analysed after T cells made contact with DCs with cytochalasin D, nocodazole, TG, or cytochalasin D and TG treatment (mean ± s.e.m., *n*=50, three independent experiments). **e** The percentage of elongated and/or flattened CD4^+^ T cells after IS formation with 1 μM, 2.5 μM, 5 μM, or 10 μM cytochalasin D treatment (mean ± s.e.m., *n*=50, three independent experiments)

To investigate the reason why the different morphologies of the T cells induced different T-cell activation states, we first studied the factors affecting T-cell morphological changes. Actin cytoskeleton rearrangements and MTOC reorientation were both involved in the T-cell morphological changes and were evoked by the TCR-mediated increase in intracellular Ca^2+^ signalling [6]. Both events provided the basis for structural remodelling and intracellular signal transduction in T cells [6]. Thus, we measured the localisation of F-actin and microtubules in T cells after IS formation. In our study, F-actin accumulated in the IS of the elongated-flattened T cells and the round-flattened T cells (Fig. 4a). However, F-actin did not accumulate in the scanning T cells, which did not become flattened and did not form an IS with DCs (data not shown). Although the fluorescence intensities of the total F-actin in the elongated-flattened T cells and the round-flattened T cells were not significantly different (data not shown), the fluorescence intensity of F-actin in the IS of the elongated-flattened T cells was lower than that in the IS of the round-flattened T cells (Fig. 4b). Moreover, the area of accumulation of F-actin in the IS of the elongated-flattened T cells was significantly smaller than that in the IS of the round-flattened T cells, consistent with the results showing that the contact area between the elongated-flattened T cells and DCs was smaller than that between the round-flattened T cells and DCs (Fig. 4c). These findings indicated that less F-actin accumulated in the IS of elongated-flattened T cells than in the IS of round-flattened T cells.

Treated with cytochalasin D, an inhibitor of F-actin polymerisation, F-actin could not accumulate in the IS formed by T cell-DC contact (Fig. 4a). Not only the percentage of CD4^+^ T cell-DC contact was reduced but also the percentages of elongated-flattened CD4^+^ T cells and round-flattened CD4^+^ T cells decreased significantly (Fig. 4d). As the dose of cytochalasin D increased, both the percentage of elongated-flattened CD4^+^ T cells and the percentage of round-flattened CD4^+^ T cells decreased. Treatment with 5 μM cytochalasin D disrupted the elongated-flattened morphology completely (Fig. 4e). These results indicated that F-actin played an important role in the formation of cell-cell contact and in morphological change of T cells. However, microtubules accumulated in the IS in 10 % of the elongated-flattened CD4^+^ T cells and in 30 % of the round-flattened CD4^+^ T cells (Additional file 7: Figure S5). After treatment with nocodazole, a microtubule inhibitor, the morphology of CD4^+^ T cells after T cell-DC contact did not change (Fig. 4d). Microtubule was not an essential factor in the morphological changes of T cell during contact with DC.

Because the morphological changes in T cells were accompanied by a Ca^2+^ response during IS formation, we investigated whether intracellular Ca^2+^ signalling could influence T-cell morphological changes. The changes in T-cell morphology were measured after treatment with thapsigargin (TG), an inhibitor of the sarco-endoplasmic Ca^2+^ ATPase that depletes Ca^2+^ stores completely and irreversibly [28], thereby leading to maximal activation of the CRAC channels and maximal Ca^2+^ release from the ER [27]. When T cells were treated with TG, the morphology of the cells remained round. When T cells were pre-treated with TG and then made contact with DCs, the percentages of T cells that became elongated-flattened and round-flattened were similar to those in the absence of TG treatment (Fig. 4d). Furthermore, we blocked F-actin polymerisation in T cells with cytochalasin D (5 μM) to abrogate the morphological changes, pre-stimulated the T cells with TG, and then added those T cells to OVA-pulsed DCs. Neither elongated-flattened nor round-flattened T cells were observed (Fig. 4d). These findings indicated that intracellular Ca^2+^ signal from Ca^2+^ stores could not affect the morphology and that the morphological changes required F-actin.

### The accumulation of F-actin in the IS contributes to the calcium influx in CD4^+^ T cells

**Fig. 5:**
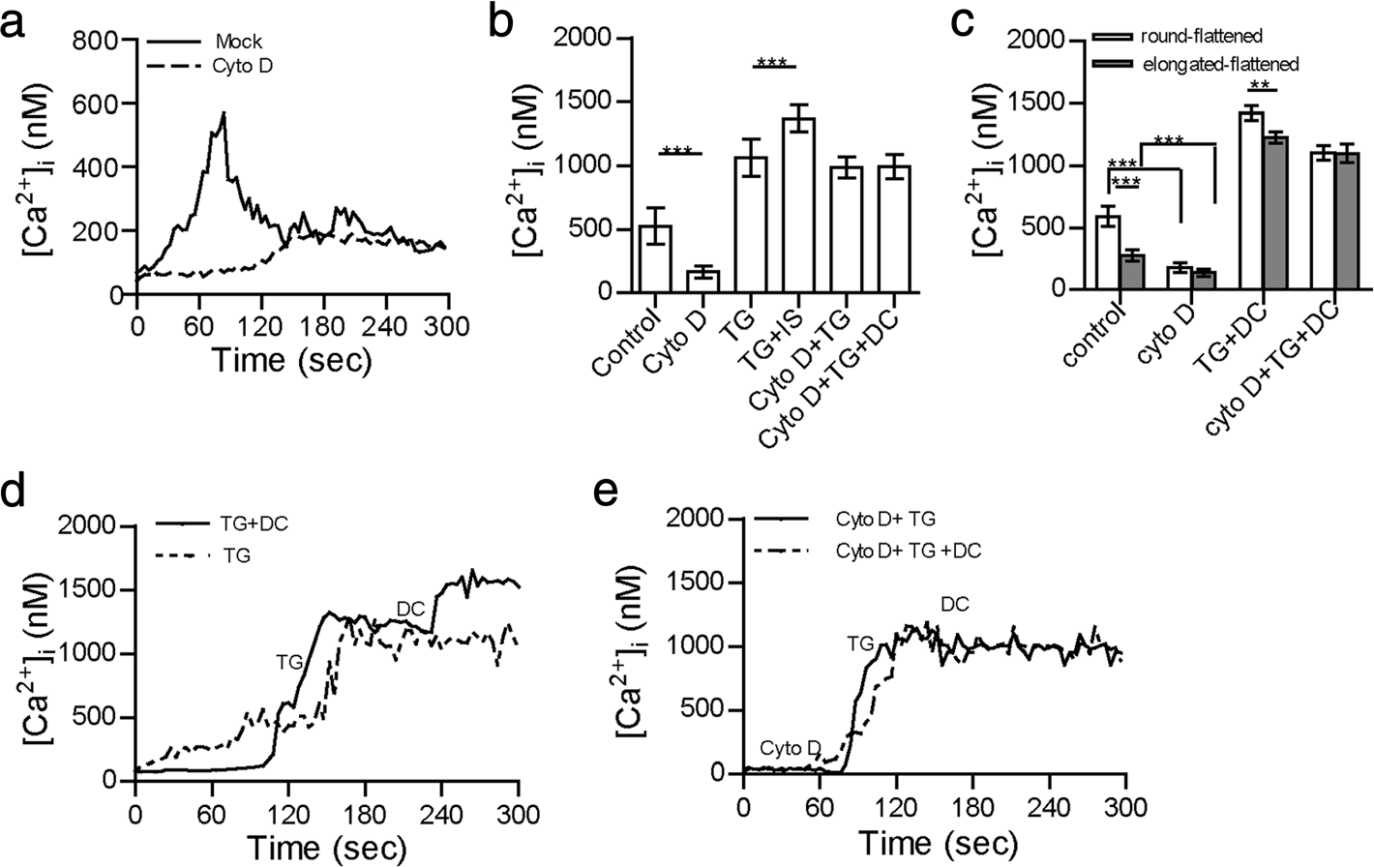
Different morphological changes in CD4^+^ T cells modulated Ca^2+^ signalling in an F-actin-dependent manner. **a** Ca^2+^ response in a CD4^+^ T cell after it made contact with a DC pulsed with OVA_323-339_ in the presence or absence of cytochalasin D. **b** Average Ca^2+^ responses were measured in CD4^+^ T cells following IS formation in the presence of cytochalasin D, nocodazole, TG or cytochalasin D and TG (mean ± s.e.m., *n*=25, three independent experiments), ***P<0.001 (two-tailed Student’s t-test). **c** Average Ca^2+^ responses were measured in elongated and/or flattened CD4^+^ T cells after they made contact with DCs in the presence of cytochalasin D or TG treatment (mean ± s.e.m., *n*=25, three independent experiments), **P<0.01, ***P<0.001 (one-way ANOVA). **d** Ca^2+^ response in a CD4^+^ T cell with TG stimulation and in a CD4^+^ T cell making contact with a DC after TG pre-treatment. **e** Ca^2+^ response in a CD4^+^ T cell with cytochalasin D and TG treatment and in a CD4^+^ T cell that made contact with a DC after cytochalasin D and TG pre-treatment. Ca^2+^ signalling was noted every 10 s

To further analyse whether F-actin participates in T-cell activation, we measured the calcium response after disrupting F-actin. Consistent with previous reports [19, 27], in the presence of cytochalasin D (1 μM), the mean peak Ca^2+^ signal and the fluctuation of the Ca^2+^ signal in CD4^+^ T cells decreased significantly (Fig. 5a-b, Additional file 6: Figure S4E-F). The mean peak Ca^2+^ signal decreased significantly not only in the round-flattened T cells but also in the elongated-flattened T cells after cytochalasin D treatment (Fig. 5c, Additional file 6: Figure S4G). These results indicated that F-actin modulated the Ca^2+^ response in CD4^+^ T cells.

Furthermore, CD4^+^ T cells were pre-treated with TG and were then co-incubated with OVA_(323–339)_-pulsed DCs. There was a greater increase of Ca^2+^ signals in the CD4^+^ T cells after IS formation (1000 nM, Fig. 5b and d, Additional file 6: Figure S4H), despite maximal depletion of the Ca^2+^ stores in the T cells before DC contact. The round-flattened T cells exhibited greater Ca^2+^ signal induction than the elongated-flattened T cells did after IS formation (Fig. 5c and Additional file 6: Figure S4G). However, with cytochalasin D pre-incubation, treatment with TG, and then contact with OVA_(323–339)_-pulsed DCs, CD4^+^ T cells did not undergo any further Ca^2+^ signal elevation (Fig. 5b and e, Additional file 6: Figure S4I). Under these conditions, there was no difference in the Ca^2+^ response between the round-flattened T cells and the elongated-flattened T cells (Fig. 5c). The results indicated that F-actin participated in the Ca^2+^ influx in the T cells.

### **F-actin modulated the** Ca^2+^**influx by controlling the distribution of calcium microdomains**

**Fig. 6:**
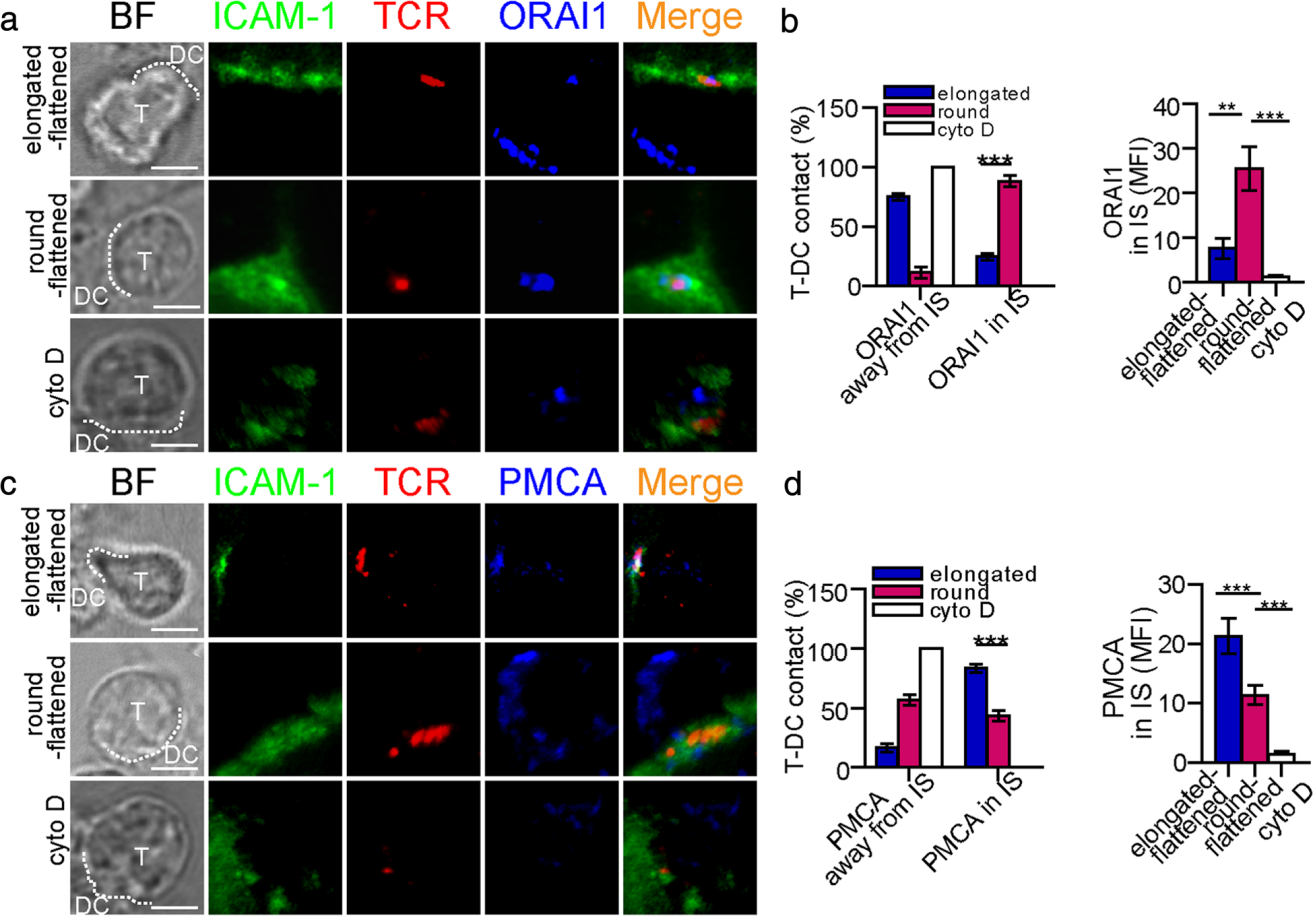
F-actin cytoskeleton-dependent morphological changes modulate Ca^2+^ signals by controlling the localisation of calcium microdomains. **a** The localisation of ORAI1 in an elongated and/or flattened CD4^+^ T cell or a CD4^+^ T cell with cytochalasin D treatment. **b** Left column: The percentage of CD4^+^ T cells with ORAI1 localisation in the IS. Right column: The MFI of ORAI1 in the IS (mean ± s.e.m., n=25, three independent experiments), **P<0.01, ***P<0.001 (two-tailed Student’s t-test). **c** The localisation of PMCA in an elongated and/or flattened CD4^+^ T cell or a CD4^+^ T cell with cytochalasin D treatment. In **a** and **c**, TCR (red) and ICAM-1 (green) are labelled to show the IS formation. The dotted white line depicts the contact boundary between the CD4^+^ T cells and the DCs. Scale bar = 2 μm. (D) Left column: The percentage of CD4^+^ T cells with PMCA localisation in the IS. Right column: The MFI of PMCA in the IS (mean ± s.e.m., *n*=25, three independent experiments), ***P<0.001 (two-tailed Student’s t-test)

There have been reports that Ca^2+^ response is correlated with the location of calcium microdomains and the movement of F-actin [19, 27]. To analyse how F-actin modulates the Ca^2+^ response in T cells, we compared the distributions of calcium microdomains in the IS between the different T-cell morphologies. ORAI1 is a key member of the CRAC channel protein family in CD4^+^ T cells. In our experiments, in 88.3 ± 4.8 % of the round-flattened CD4^+^ T cells, ORAI1 localised in the synapse or puncta (near the site of stimulation). In 75 ± 2.87 % of the elongated-flattened CD4^+^ T cells, ORAI1 was away from the IS (Fig. 6a-b). The fluorescence intensity of ORAI1 in the IS of the elongated-flattened CD4^+^ T cells was lower than that in the IS of the flattened CD4^+^ T cells (Fig. 6b, right column).

PMCA is the major mediator of Ca^2+^ extrusion from T cells [29]. PMCA4b, a PMCA subtype, is highly expressed in T cells [29]. We analysed the localisation of PMCA4b in CD4^+^ T cells during IS formation. In 83.3 ± 3.5 % of the elongated-flattened T cells, PMCA localised in the synapse. However, in 43.3 ± 4.5 % of the round-flattened T cells, PMCA localised in the synapse. The fluorescence intensity of PMCA in the synapse of the elongated-flattened T cells was higher than that in the IS of the round-flattened T cells (Fig. 6c-d).

**Fig. 7:**
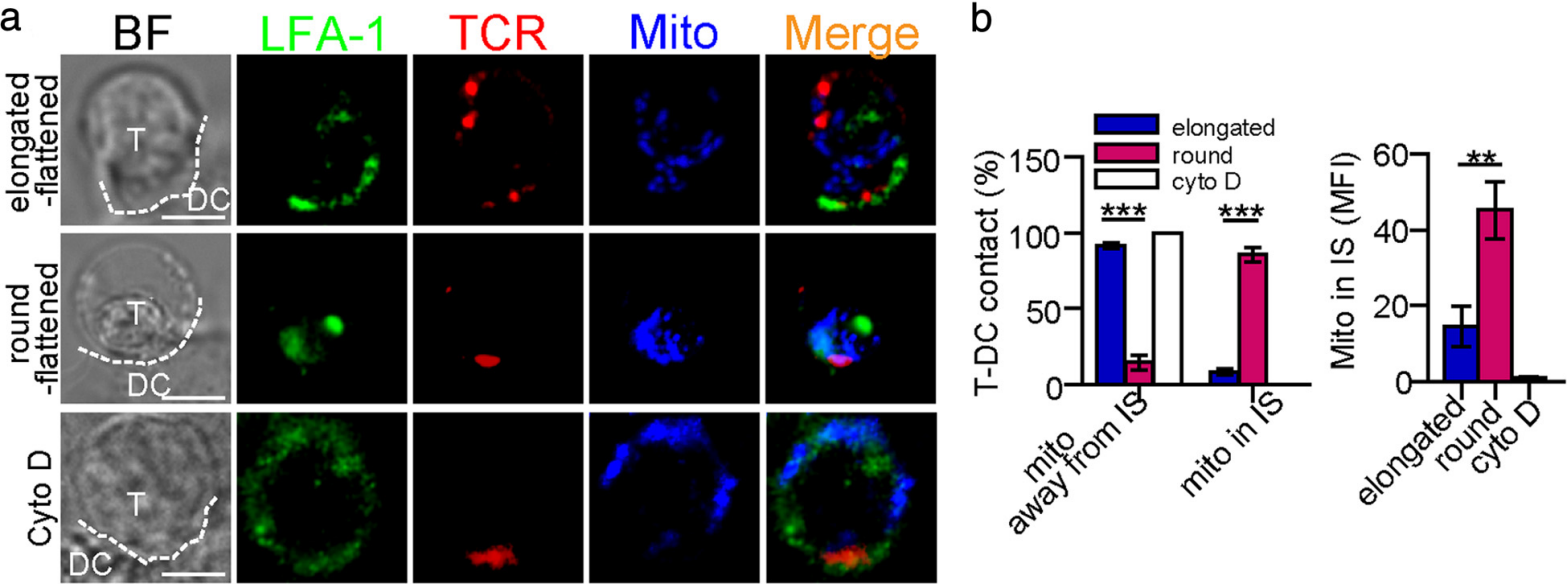
F-actin modulated the distribution of mitochondria. **a** The localisation of mitochondria (blue) in an elongated and/or flattened CD4^+^ or a CD4^+^ T cell with cytochalasin D treatment. TCR (red) and LFA-1 (green) were labelled to show the IS formation. The dotted white line depicts the contact boundary between the CD4^+^ T cells and the DCs. Scale bar = 2 μm. **b** Left column: The percentage of CD4^+^ T cells with mitochondrial localisation in the IS. Right column: The MFI of mitochondria in the IS in CD4^+^ T cells (mean ± s.e.m., n=25, three independent experiments), **P<0.01, ***P<0.001 (two-tailed Student’s t-test)

Mitochondria play an important role in Ca^2+^ homeostasis in T cells and in mediation of the localisation of calcium microdomains in the IS [19]. In our experiments, mitochondria were distributed at synaptic sites in 85.6 ± 4.7 % of round-flattened T cells, whereas mitochondria were only found at synaptic sites in 8.3 ± 1.8 % of elongated-flattened T cells. The MFI of the mitochondria in the IS of the round-flattened T cells was higher than that in the IS in the elongated-flattened T cells (Fig. 7a-b).

With cytochalasin D treatment, there was no accumulation of ORAI1 or PMCA in the synapses of the T cells (Fig. 6a and c). The fluorescence intensities of ORAI1 and PMCA in the IS decreased significantly (Fig. 6b and d). Moreover, with cytochalasin D treatment, mitochondria were not present in the synapses of CD4^+^ T cells (Fig. 7a). The fluorescence intensity of the mitochondria in the IS decreased significantly (Fig. 7b). These data indicated that F-actin modulated the distribution of calcium microdomains to be localised in or near the IS. The different distributions of calcium microdomains between elongated-flattened T cells and round-flattened T cells indicated that the different Ca^2+^ signals between the two T-cell morphologies may be modulated by the amount of F-actin accumulation in the IS.

## Discussion

T-cell polarisation accompanied by altered morphology is important for T-cell activation [8, 10, 11]. Spherical T cells have been reported to become elongated and/or flattened after contact with an APC [8, 10]. In contrast to contact with B cells, T cells contact with DCs led to diverse T-cell morphological changes in our experiments. Especially, the round-flattened shape change was mostly observed in T cell during contact with DC. Only T cells that became flattened, undergoing either round-flattened or elongated-flattened morphological changes, formed an IS with DCs. There are three characteristics that differ between the round-flattened morphology and the elongated-flattened morphology. First, the recognition phase of the round-flattened T cell is shorter than that of the elongated-flattened T cell. Second, the range of the shape index of round-flattened T cell is narrower than that of the elongated-flattened T cell (1–1.5 vs 1–2.5). Third, the contact areas formed by round-flattened T cell-DC contact are larger than those formed by elongated-flattened T cell-DC contact. These phenomena indicated that cytoskeletal polymerisation was different between these two T-cell shapes, which may lead to different T-cell activation states.

The relationship between morphological changes and T-cell activation has attracted much attention, but the relationship between morphological changes and the activation of naive T cells in contact with DCs is rarely described. The diversity of T-cell morphological changes during T cell-DC contact may lead to diverse T-cell activation states. Here, we found that only flattening of a T cell, including elongated-flattened morphology and round-flattened morphology, correlated with Ca^2+^ release in the T cell. The vast majority of T cells became round-flattened when they made contact with DCs, whereas minority became elongated-flattened. Only the round-flattened T cells exhibited a high-level Ca^2+^ response and the accumulation of activated TCR signalling molecules in the IS. In contrast, the elongated-flattened T cells exhibited a low-level Ca^2+^ response and did not accumulate TCR signalling molecules in the IS. A sustained high level of Ca^2+^ signalling can sufficiently induce T-cell activation, whereas a low level of Ca^2+^ signalling for a long period of time may induce T-cell tolerance [21]. The round-flattened T cells may be activated efficiently, whereas the elongated-flattened T cells may fail to be activated. Although T-cell polarisation is helpful for T-cell activation, excessive tension may be not efficient for T-cell activation. In addition, round-flattened shape of T cell was different from T cell contacted B cell but occupied the major proportion of T cell during contact with DC, and was activated efficiently. These results may explain why DCs can activate more antigen-specific T cells than B cells in a short period of time. In our observation, we found that elongated-flattened change and round-flattened change of T cell occurred before IS formation (IS was formed at 1,800 s in Fig. 2a and at 600 s in Fig. 2b) and Ca^2+^ release (at 600 s in Fig. 2a and at 300 s in Fig. 2b). Due to IS formation controlled T-cell activation [1], morphological change before IS formation is an initial factor for T-cell activation. For the T cell without elongated-flattened or round-flattened shape change, T cell can not be activated (Fig. 2c). When disrupting the morphology of T cells with cytochalasin D, T-cell activation was decreased (Fig. 4a-b). Thus the morphological changes of T cell modulate T-cell activation. To investigate whether different T cell activation status determines their morphology changes during DC activation, we obsevered the morphology of T cell and found that it was changed after T cell was activated. The elongated-flattened T cell changed to round-flattened after IS formation for ten minutes (Additional file 8: Figure S6 A-B) while the round-flattened T cell did not. It indicated that T-cell activation might affect the morphological change of T cell in return. In this manuscript, we focus on how the morphological change modulates T-cell activation. The investigation how T-cell activation affects the morphological change of T cell would be our further study.

Furthermore, we have shown that F-actin plays an important role in the process of morphological changes modulating T-cell activation. Moreover, greater accumulation of F-actin in the IS is better for T-cell activation. Previously, F-actin was shown to play at least three important roles in antigen recognition: (1) forming the IS by organising distinct supramolecular activation clusters through actin cytoskeleton rearrangements [1, 30]; (2) involving signalling complexes that are dependent on a scaffold of actin filaments [8, 31–33]; and (3) modulating Ca^2+^ influx by controlling the spatial and temporal distribution of Ca^2+^ sources and sinks [24, 34, 35]. In our experiments, we found that the intensity of F-actin accumulation in the IS correlates with the strength of T-cell activation. In round-flattened T cells, more F-actin accumulation in the IS is better for stable synapse formation and organisation of the supermolecular activation clusters involving ZAP-70, PLC-γ, PKC-θ, which further activate the TCR signalling pathway to trigger a calcium response. A large amount of F-actin is polarised to the IS to bring more mitochondria to localise into the IS. Previous reports showed the localisation of mitochondria in the IS prevent Ca^2+^-dependent channel inactivation and sustain the activity of the CRAC channels for a long period to allow a prolonged Ca^2+^ influx, which enhances Ca^2+^-dependent T-cell activation and subsequent proliferation [19, 20]. Therefore, a large amount of F-actin in IS will be of great benefit to T-cell activation by bringing more mitochondria close to the IS. However, in elongated-flattened T cells, less accumulation of F-actin reduces its ability to bring a large proportion of the mitochondria close to the IS. In case mitochondrial accumulation at the IS is prevented, Ca^2+^ accumulates in a microdomain close to the CRAC/ORAI channels, which leads to inactivation of the channels and efficient Ca^2+^ clearance by PMCA [24, 27]. These events result in a sustained low Ca^2+^ signal in elongated-flattened T cells, which is consistent with a report by Ariel Quintana [20]. In addition, TG treatment can lead to maximal activation of CRAC channels, but had no impact on the TCR signaling molecules, cytoskeletal proteins, morphological change and the localization of ORAI1 [36]. Therefore, the activation of CRAC channels could not affect morphological change. After IS formation, TCR signaling molecules, cytoskeletal proteins and ORAI1 were activated. The localization of ORAI1 in CRAC channels in IS were correlated with different morphological T cells. Due to cytoskeletal protein was reported to affect the localization of ORAI1 in IS [37, 38]. The cytoskeletal proteins affected morphological change of T cells, while ORAI1 in CRAC did not. Therefore, in T cells with different morphologies, different T-cell activation states may be attributable to the ability of F-actin to accumulate in the IS, which leads to different distributions of calcium microdomains and levels of Ca^2+^ release.

In addition, antigen dose could not affect the ratio of elongated-flattened T cells to round-flattened T cells but decreased the numbers of elongated-flattened T cells and round-flattened T cells. It indicated elongated or flattened shape change is not correlated with antigen dose. The reason why elongated-flattened morphology is associated with low activation of T cells whereas the round-flattened morphology is associated with effective T cell activation may be the different expressions of membrane surface molecules on these two types of T cells. Some membrane surface molecules are associated with F-actin that affect morphological change. These molecules may also be associated with T-cell activation. For example, CTLA-4 or phosphatidylinositol 3-kinase may participate in morphological change and T-cell activation [39–42]. In addition, some adhesion molecules may participate in morphological change and T-cell activation. For example, LFA-1 is reported to accumulate into T-DC contact before TCR accumulation and affect the movement of F-actin that is associated with morphological change [1, 42]. Moreover, LFA-1 is an important molecule for IS formation and T-cell activation. It is worthy to investigate which molecules regulate morphological change and the activation of T cell. The elongated morphological change in T cells is reported to be mediated by CTLA-4 [40] and CD44 [41]. Both of these molecules are dependent on phosphatidylinositol 3-kinase, Vav-1, Cdc42, myosin light chain kinase, Src-family kinases, and phospholipase C [40, 41] to further affect F-actin polymerisation. Elongated T cells may be a subset that express specific adhesion molecules to mediate cell-cell contact, F-actin polymerisation and higher-order F-actin bundling [43]. Therefore, the different morphological change may occurr in two different subsets of CD4^+^ T cells. What adhesion molecules are essential for shape changes and what the function of such a CD4^+^ T cell subset with special adhesion molecules will be the subjects of our future studies.

## Conclusion

In conclusion, we found that F-actin accumulation in the IS participates in the processes of T-cell morphological changes and activation and modulates Ca^2+^ influx by controlling the distribution of calcium microdomains. Not all antigen-specific T cells are activated to release a high level of calcium signal; only round-flattened T cells accumulate a large amount of F-actin in the IS and exhibit a high-level Ca^2+^ response. Other T cells, which become elongated-flattened, exhibit a low-level calcium response. Loss of the ability of F-actin to accumulate in the IS may lead to hyporesponsiveness among elongated-flattened T cells, which may play a regulatory role in T-cell activation. The mechanism and the function of such hyporesponsive T cells should be studied further.

## Additional files

Additional file 1: Figure S1. Morphological changes in the scanning CD4^+^ T cells (A) *Left panel*: A round CD4^+^ T cell before and after scanning DC. TCRs were labelled with H57-597-Fab- Alexa 647 (red), while ICAM-1 was fused with EGFP (green). *Right panel*: Grey value profiles along the red and black lines. (B) The shape index change and Ca^2+^ signal in a round CD4^+^ T cell which scanned OVA_(323–339)_-pulsed DC. (C) An elongated CD4^+^ T cell scanning DC (left panel) and the grey value analysis along the line (right panel). (D) The shape index change and Ca^2+^ signal in an elongated T cell which scanned OVA_(323–339)_-pulsed DC. (E) The shape index of elongated and/or flattened scanning CD4^+^ T cells (*n* = 25, the data are shown as the mean ± s.e.m.). Additional file 2: Figure S2. The morphological change in CD4^+^ T cells during contacted with DC. (A) The T cell became elongated-flattened after contacting DC. The T cell before contacting DC (left) and after contacting DC (right) was shown. The arrow pointed to the T cell is the same T cell in Fig. 1a. (B) The T cell became round-flattened after contacting DC. The T cell before contacting DC (left) and after contacting DC (right) was shown. The arrow pointed to the T cell is the same T cell in Fig. 1b. Additional file 3: Video 1. The morphology of a CD4^+^ T cell changes from round to enlongated-flattened during the IS formation. TCR is shown red, ICAM-1 is green, and scale bar is 2 μm. Before IS formation, TCR was uniformed on the surface of T cell. After IS formation, TCR and ICAM-1 was accumulated at the interface of T-DC. Additional file 4: Video 2. The morphology of a CD4^+^ T cell changes from round to round-flattened during the IS formation. TCR is shown red, ICAM-1 is green, and scale bar is 2 μm. Before IS formation, TCR was uniformed on the surface of T cell. After IS formation, TCR and ICAM-1 was accumulated at the interface of T-DC. Additional file 5: Figure S3. Under different OVA_(323–339)_ doses, the percentage of elongated-flattened and round-flattened T cells among the CD4^+^ T cells contacting DCs. (the data are shown as the mean ± s.e.m., *n* = 60, from three independent experiments). Additional file 6: Figure S4. Ca^2+^ responses in CD4^+^ T cells were measured and presented by △F/F. (A) The shape index change and Ca^2+^ signal in a CD4^+^ T cell whose morphology changed to elongated-flattened (top panel). (B) The shape index change and Ca^2+^ signal of a CD4^+^ T cell whose morphology changed to flattened (top panel). (C) The shape index change and Ca^2+^ signal in a resting T cell. (A-C) Ca^2+^ signalling was obtained every 10-s or 40-s. (D) Average Ca^2+^ responses of CD4^+^ T cells whose morphology changed to round-flattened or to elongated-flattened. (Data are shown as mean ± s.e.m., two-tailed Student’s *t*-test, ****p* < 0.001). (E) Ca^2+^ response of a CD4^+^ T cell after it contacted DC pulsed OVA_(323–339)_ in the presence of the cytochalasin D or not. (F) Average Ca^2+^ responses were measured in CD4^+^ T cells during the IS formation in the presence of cytochalasin D, nocodazole, TG or cytochalasin D and TG. (mean ± s.e.m, *n* = 25, three independent experiments), ****P* < 0.001 (two-tailed Student’s *t*-test). (G) Average Ca^2+^ responses were measured in elongated and/ or CD4^+^ T cells during contact with DC in the presence of the cytochalasin D or TG treatment. (mean ± s.e.m, *n* = 25, three independent experiments), ** *P* < 0.01, ****P* < 0.001 (two-tailed Student’s *t*-test). (H) Ca^2+^ response in a CD4^+^ T cell with TG stimulation and in a CD4^+^ T cell forming IS with TG pretreatment. (I) Ca^2+^ response of a CD4^+^ T cell with the cytochalasin D and TG treatment and a CD4^+^ T cells that formed IS with the cytochalasin D and TG pretreatment. Additional file 7: Figure S5. The distribution of microtubules in CD4^+^ T cells which made contact with DCs. The distribution of microtubules was measured in an elongated-flattened CD4^+^ T cell (top line) or a round-flattened CD4^+^ T cell (middle line). In the presence of the nocodazole, the distribution of microtubules was measured in a CD4^+^ T cell (bottom line). TCR (red) and ICAM-1 (green) are used to mark the structure of IS, respectively. Dotted white line depicts the contact boundary of CD4^+^ T cells and DCs. Scale bar is 2 μm. Additional file 8: Figure S6. The relationship between morphological changes and T-cell activation before and after IS formation. (A) Shape index changes and Ca^2+^ signals in a CD4^+^ T cell whose morphology changed to elongated-flattened (left panel). At 1,080 s, IS between CD4^+^ T cell and DC was formed. Before IS formation, the morphology of CD4^+^ T cell changed from round to elongated-flattened. At 1,680 s, the morphology of CD4^+^ T cell changed from elongated-flattened to round-flattened. The peak of Ca^2+^ signal was occurred before IS formation (at 800 s) and Ca^2+^ signal sustained at a low level. Images of the morphology and Ca^2+^ signals of the elongated-flattened T cell before and after IS formations are shown in the right panel. (B) Shape index changes and Ca^2+^ signals in a CD4^+^ T cell whose morphology changed to flattened (left panel). Images of the morphology and Ca^2+^ signals of the round-flattened T cell are shown in the right panel. IS between T cell and DC was formed at 560 s, when Ca^2+^ signal was at the highest level. The dotted white line depicts the contact boundary between the OT-II CD4^+^ T cells and the DCs. The calcium intensity was pseudo-colored with hues ranging from blue (low) to red (high). Ca^2+^ signalling was obtained every 40 s. ICAM-1 was labelled to be green. TCR was labelled to be red. After IS formation, TCR and ICAM-1 were accumulated into the IS of DC-T. Scale bar = 2 μm. (C-E) The distribution of ZAP-70, PLC-γ, PKC-θ (blue) and TCR (red) in the resting CD4^+^ T cell are shown in panel C to E, reseparately. Scale bar = 2 μm.

## Abbreviations

IS: Immunological synapse
DC: Dendritic cell
TCR: T-cell receptor
ZAP-70: Zeta-chain-associated protein kinase 70
PLC-γ: Phospholipase C-γ
PKC-θ: Protein kinase C-θ
APC: Antigen-presenting cell
c-SMAC: Central supramolecular activation cluster
MTOC: Microtubule-organising centre
ER: Endoplasmic reticulum
CRAC: Ca^2+^ release-activated Ca^2+^ channels
LAT: Linker of activated T cells
PMCAs: Plasma membrane Ca^2+^ ATPases
F-actin: Filamentous actin
BF: Bright-field microscopy
MFI: Mean fluorescence intensity
