# Supplementary figures and images for: Morphological change of CD4+ T cell during contact with DC modulates T-cell activation by accumulation of F-actin in the immunology synapse
Source: BMC Immunol. 2015 Aug 26;16:49. doi: 10.1186/s12865-015-0108-x (PMC4549951; doi:10.1186/s12865-015-0108-x)

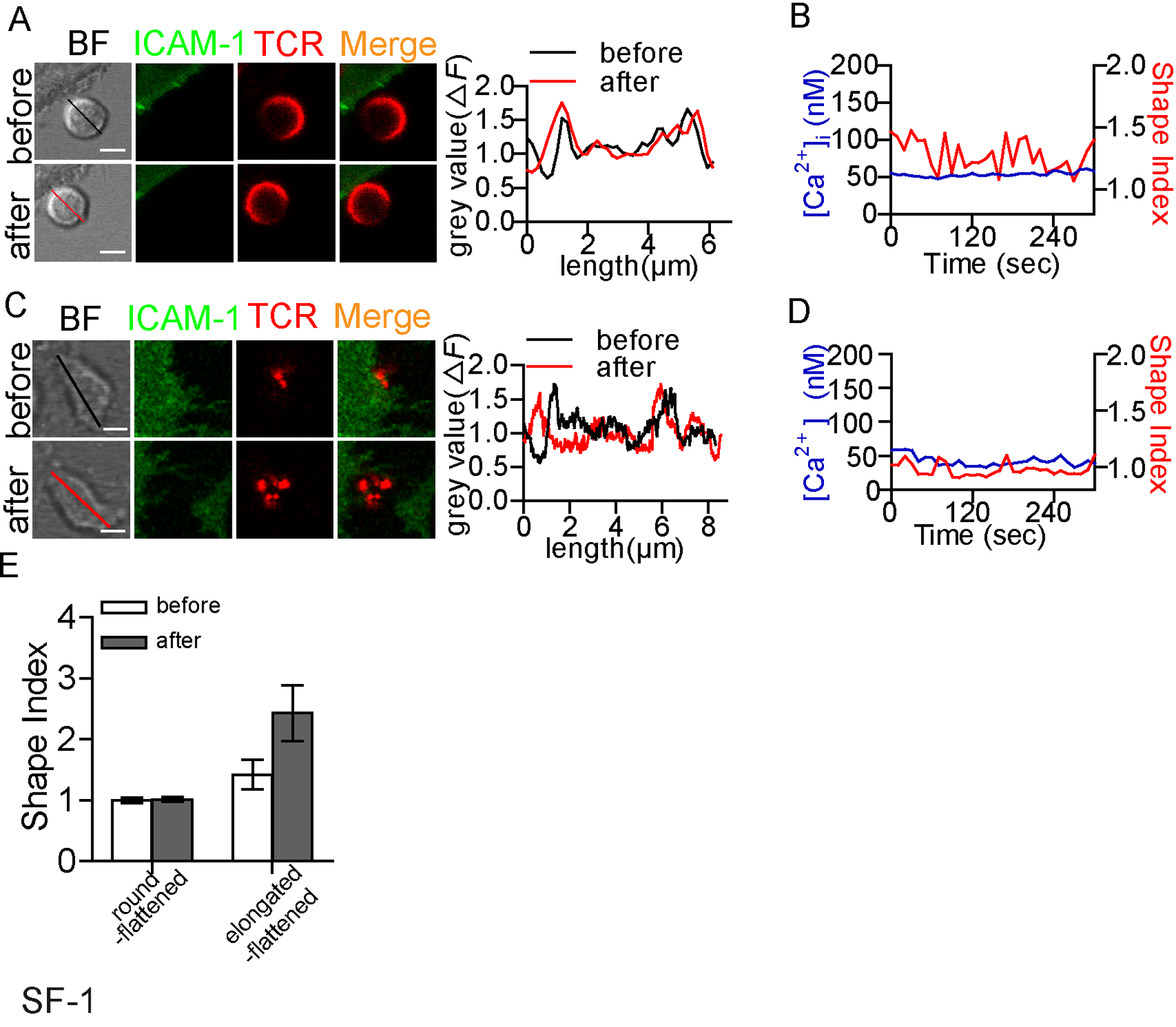

Supplement: Additional file 1: Figure S1. — Morphological changes in the scanning CD4+ T cells (A) Left panel: A round CD4+ T cell before and after scanning DC. TCRs were labelled with H57-597-Fab- Alexa 647 (red), while ICAM-1 was fused with EGFP (green). Right panel: Grey value profiles along the red and black lines. (B) The shape index change and Ca2+ signal in a round CD4+ T cell which scanned OVA(323–339)-pulsed DC. (C) An elongated CD4+ T cell scanning DC (left panel) and the grey value analysis along the line (right panel). (D) The shape index change and Ca2+ signal in an elongated T cell which scanned OVA(323–339)-pulsed DC. (E) The shape index of elongated and/or flattened scanning CD4+ T cells (n = 25, the data are shown as the mean ± s.e.m.). [file 12865_2015_108_MOESM1_ESM.tif]

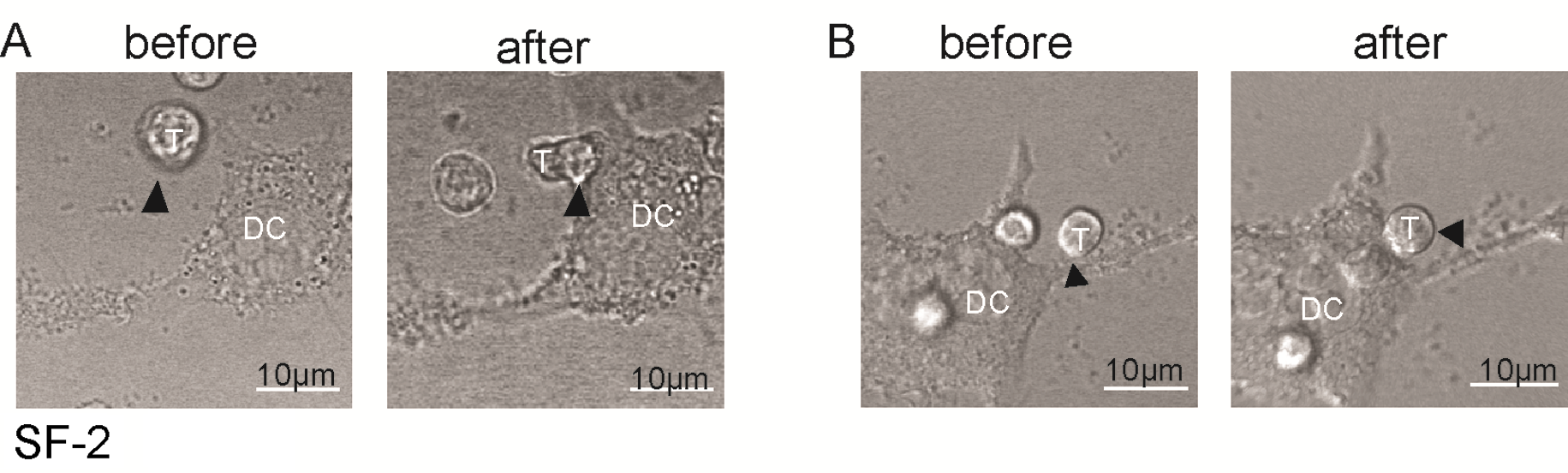

Supplement: Additional file 2: Figure S2. — The morphological change in CD4+ T cells during contacted with DC. (A) The T cell became elongated-flattened after contacting DC. The T cell before contacting DC (left) and after contacting DC (right) was shown. The arrow pointed to the T cell is the same T cell in Fig. 1a. (B) The T cell became round-flattened after contacting DC. The T cell before contacting DC (left) and after contacting DC (right) was shown. The arrow pointed to the T cell is the same T cell in Fig. 1b. [file 12865_2015_108_MOESM2_ESM.tif]

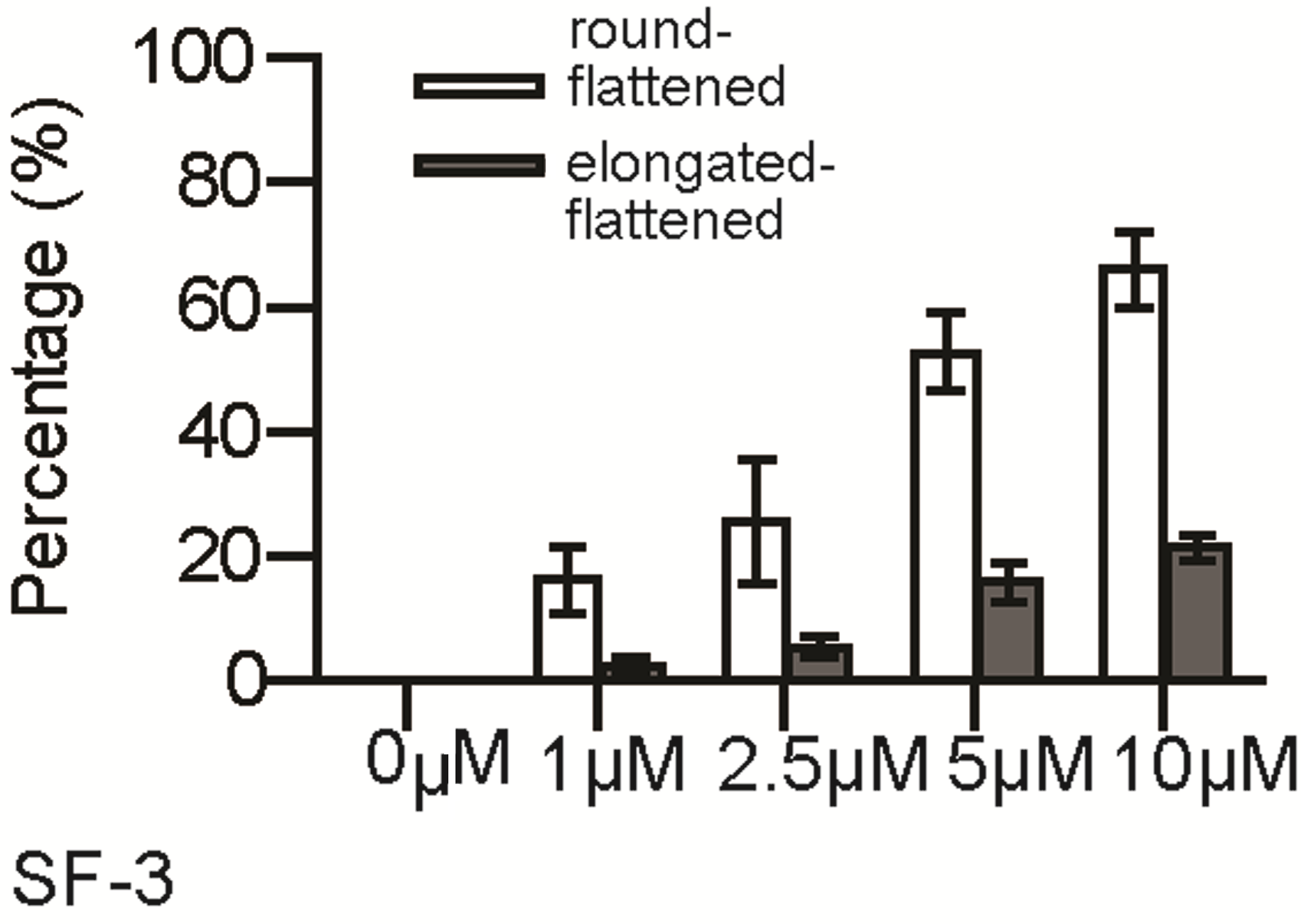

Supplement: Additional file 5: Figure S3. — Under different OVA(323–339) doses, the percentage of elongated-flattened and round-flattened T cells among the CD4+ T cells contacting DCs. (the data are shown as the mean ± s.e.m., n = 60, from three independent experiments). [file 12865_2015_108_MOESM5_ESM.tif]

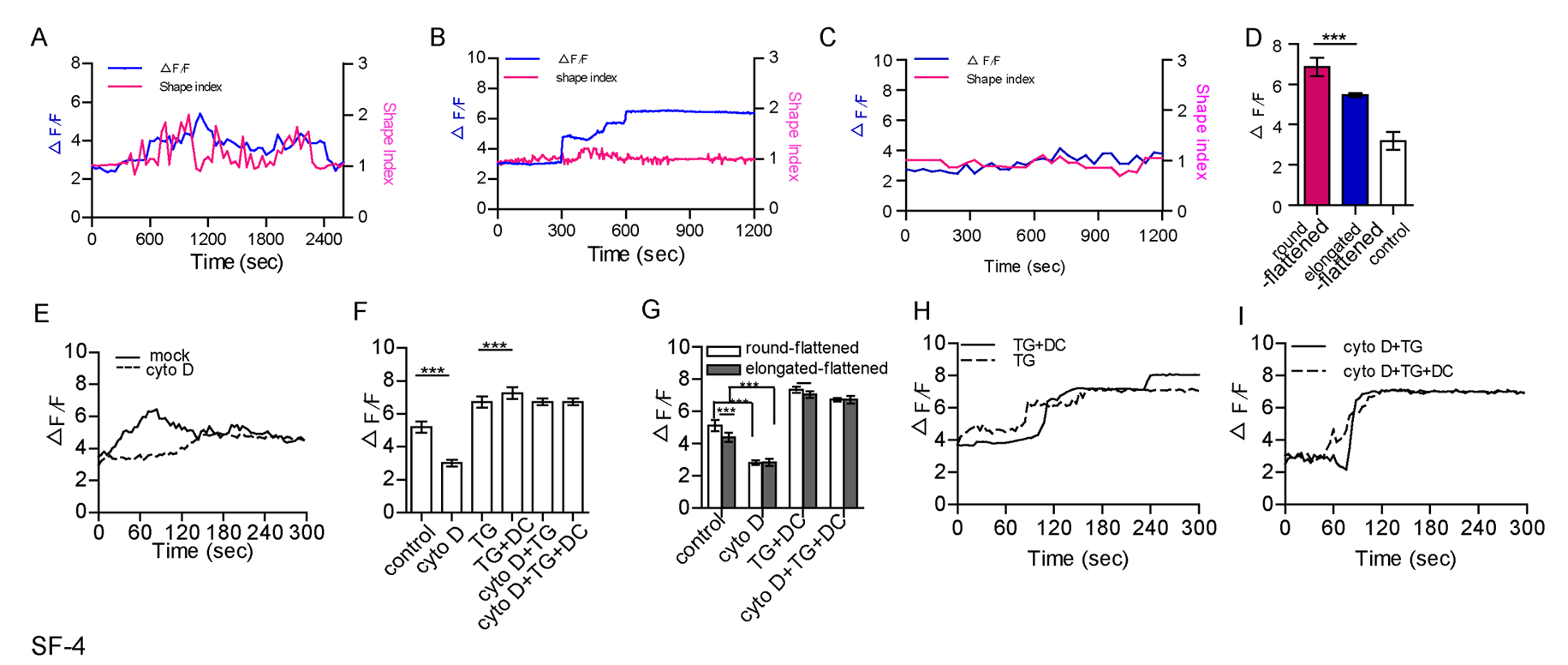

Supplement: Additional file 6: Figure S4. — Ca2+ responses in CD4+ T cells were measured and presented by △F/F. (A) The shape index change and Ca2+ signal in a CD4+ T cell whose morphology changed to elongated-flattened (top panel). (B) The shape index change and Ca2+ signal of a CD4+ T cell whose morphology changed to flattened (top panel). (C) The shape index change and Ca2+ signal in a resting T cell. (A-C) Ca2+ signalling was obtained every 10-s or 40-s. (D) Average Ca2+ responses of CD4+ T cells whose morphology changed to round-flattened or to elongated-flattened. (Data are shown as mean ± s.e.m., two-tailed Student’s t-test, ***p < 0.001). (E) Ca2+ response of a CD4+ T cell after it contacted DC pulsed OVA(323–339) in the presence of the cytochalasin D or not. (F) Average Ca2+ responses were measured in CD4+ T cells during the IS formation in the presence of cytochalasin D, nocodazole, TG or cytochalasin D and TG. (mean ± s.e.m, n = 25, three independent experiments), ***P < 0.001 (two-tailed Student’s t-test). (G) Average Ca2+ responses were measured in elongated and/ or CD4+ T cells during contact with DC in the presence of the cytochalasin D or TG treatment. (mean ± s.e.m, n = 25, three independent experiments), ** P < 0.01, ***P < 0.001 (two-tailed Student’s t-test). (H) Ca2+ response in a CD4+ T cell with TG stimulation and in a CD4+ T cell forming IS with TG pretreatment. (I) Ca2+ response of a CD4+ T cell with the cytochalasin D and TG treatment and a CD4+ T cells that formed IS with the cytochalasin D and TG pretreatment. [file 12865_2015_108_MOESM6_ESM.tif]

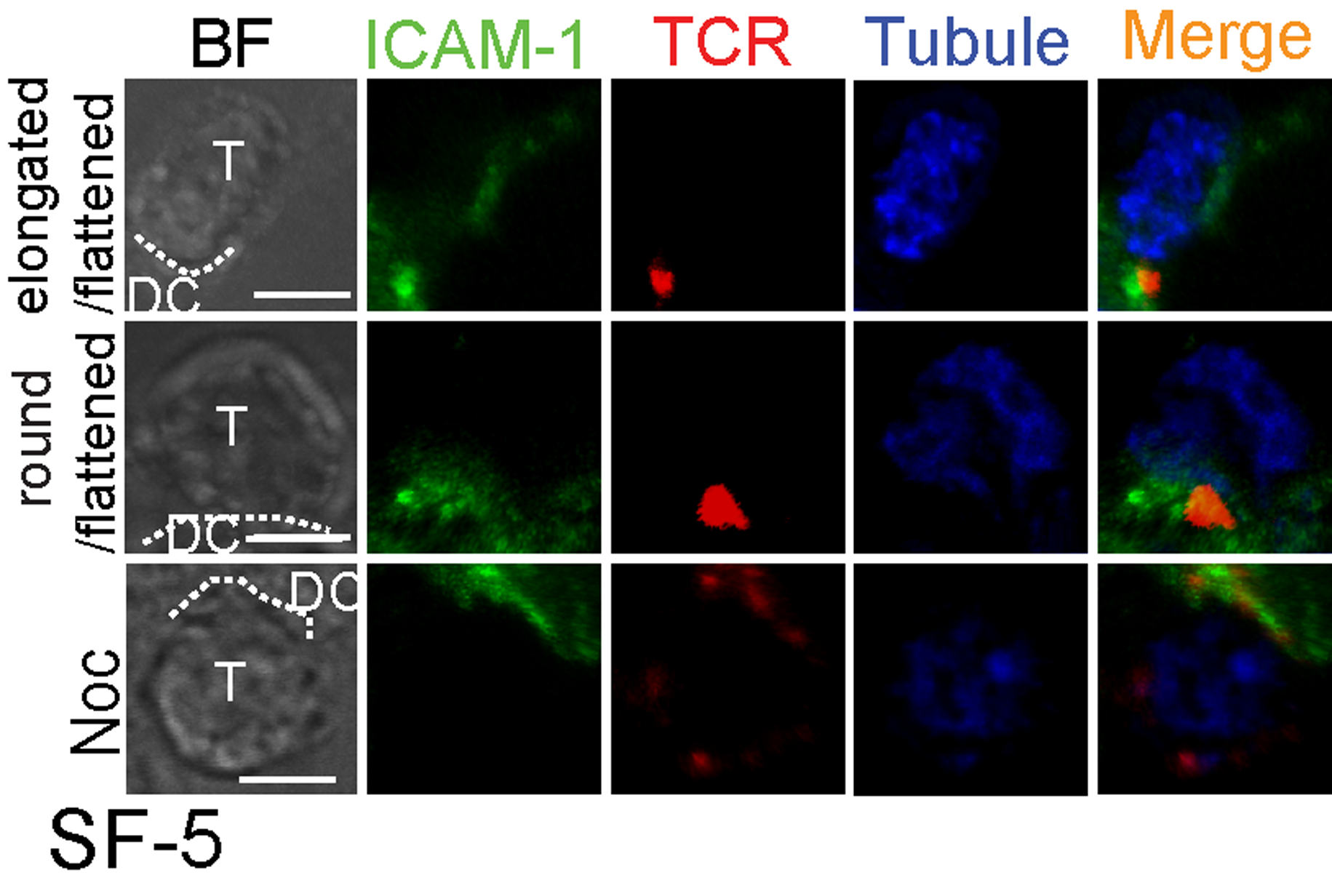

Supplement: Additional file 7: Figure S5. — The distribution of microtubules in CD4+ T cells which made contact with DCs. The distribution of microtubules was measured in an elongated-flattened CD4+ T cell (top line) or a round-flattened CD4+ T cell (middle line). In the presence of the nocodazole, the distribution of microtubules was measured in a CD4+ T cell (bottom line). TCR (red) and ICAM-1 (green) are used to mark the structure of IS, respectively. Dotted white line depicts the contact boundary of CD4+ T cells and DCs. Scale bar is 2 μm. [file 12865_2015_108_MOESM7_ESM.tif]

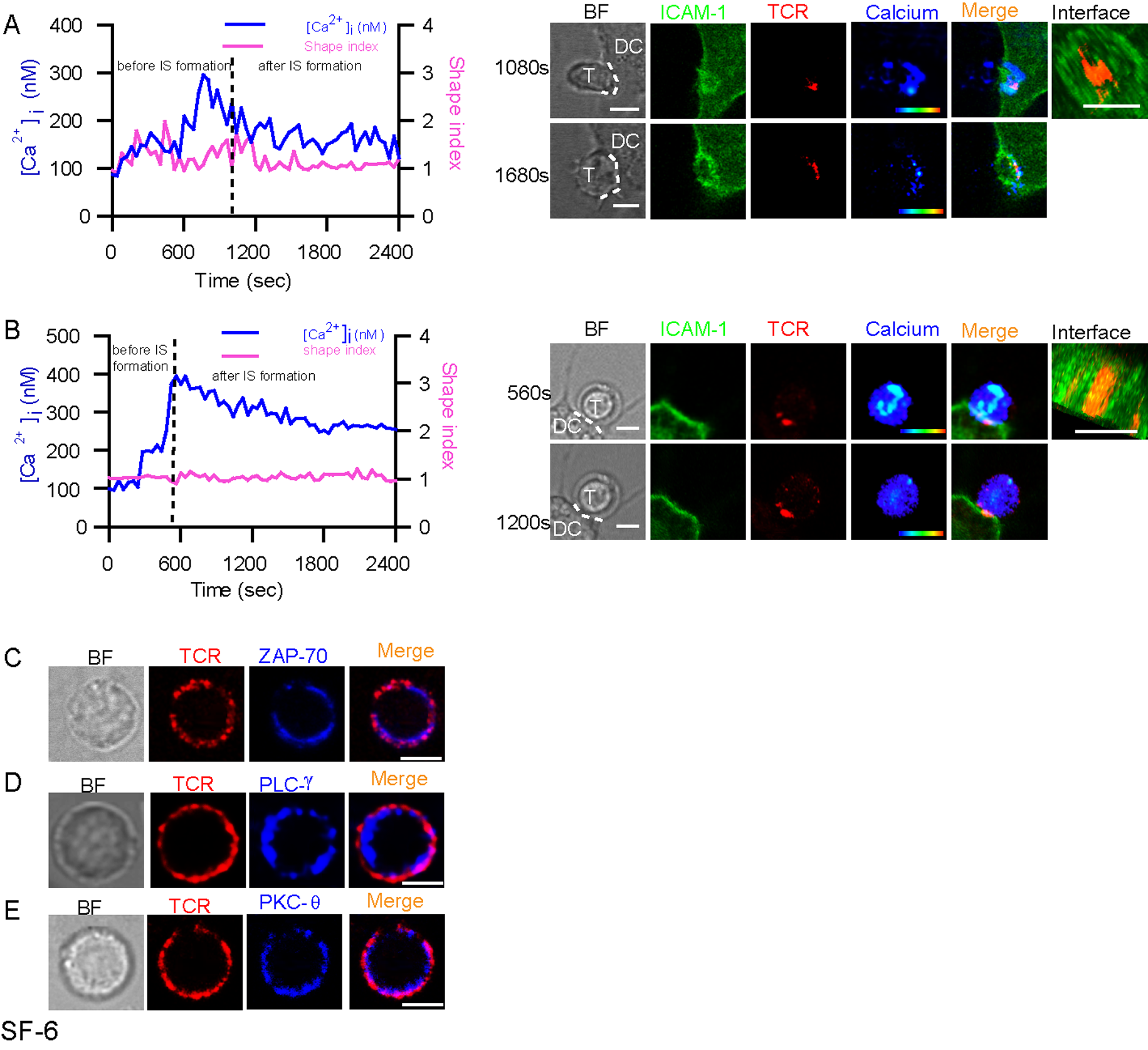

Supplement: Additional file 8: Figure S6. — The relationship between morphological changes and T-cell activation before and after IS formation. (A) Shape index changes and Ca2+ signals in a CD4+ T cell whose morphology changed to elongated-flattened (left panel). At 1,080 s, IS between CD4+ T cell and DC was formed. Before IS formation, the morphology of CD4+ T cell changed from round to elongated-flattened. At 1,680 s, the morphology of CD4+ T cell changed from elongated-flattened to round-flattened. The peak of Ca2+ signal was occurred before IS formation (at 800 s) and Ca2+ signal sustained at a low level. Images of the morphology and Ca2+ signals of the elongated-flattened T cell before and after IS formations are shown in the right panel. (B) Shape index changes and Ca2+ signals in a CD4+ T cell whose morphology changed to flattened (left panel). Images of the morphology and Ca2+ signals of the round-flattened T cell are shown in the right panel. IS between T cell and DC was formed at 560 s, when Ca2+ signal was at the highest level. The dotted white line depicts the contact boundary between the OT-II CD4+ T cells and the DCs. The calcium intensity was pseudo-colored with hues ranging from blue (low) to red (high). Ca2+ signalling was obtained every 40 s. ICAM-1 was labelled to be green. TCR was labelled to be red. After IS formation, TCR and ICAM-1 were accumulated into the IS of DC-T. Scale bar = 2 μm. (C-E) The distribution of ZAP-70, PLC-γ, PKC-θ (blue) and TCR (red) in the resting CD4+ T cell are shown in panel C to E, reseparately. Scale bar = 2 μm. [file 12865_2015_108_MOESM8_ESM.tif]
